# Fatty acid analysis as a tool to infer the diet in Illinois river otters (*Lontra canadensis*)

**DOI:** 10.1186/2055-0391-56-16

**Published:** 2014-09-02

**Authors:** Damian Satterthwaite-Phillips, Jan Novakofski, Nohra Mateus-Pinilla

**Affiliations:** Illinois Natural History Survey, University of Illinois Urbana-Champaign, 1816 S. Oak Street, Champaign, IL 61820 USA; Department of Animal Sciences, University of Illinois at Urbana-Champaign, 1503 S. Maryland Drive, Urbana, IL 61801 USA

**Keywords:** River otter (*Lontra canadensis*), Inferring diet, Quantitative fatty acid signature analysis (QFASA), Predation, Gas chromatography, Principal components analysis (PCA)

## Abstract

Fatty acids (FA) have recently been used in several studies to infer the diet in a number of species. While these studies have been largely successful, most have dealt with predators that have a fairly specialized diet. In this paper, we used FA analysis as a tool to infer the diet of the nearctic river otter (*Lontra canadensis*). The river otter is an opportunistic predator known to subsist on a wide variety of prey including, fishes, crayfish, molluscs, reptiles and amphibians, among others. We analyzed the principle components of 60 FA from otters and 25 potential prey species in Illinois, USA. Prey species came from 4 major taxonomic divisions: fishes, crayfish, molluscs and amphibians. Within each division, most, but not all, species had significantly different profiles. Using quantitative FA signature analysis, our results suggest that, by mass, fish species are the most significant component of Illinois River otters’ diet (37.7 ± 1.0%). Molluscs ranked second (32.0 ± 0.8%), followed by amphibians (27.3 ± 4.3%), and finally, crayfish (3.0 ± 0.6%). Our analysis indicates that molluscs make up a larger portion of the otter diet than previously reported. Throughout much of the Midwest there have been numerous otter reintroduction efforts, many of which appear to be successful. In regions where mollusc species are endangered, these data are essential for management agencies to better understand the potential impact of otters on these species. Our analysis further suggests that quantitative FA signature analysis can be used to infer diet even when prey species are diverse, to the extent that their FA profiles differ. Better understanding of the otter’s metabolism of FA would improve inferences of diet from FA analysis.

## Background

In Illinois, and throughout the Midwestern region of the United States, river otter (*Lontra canadensis*) reintroduction programs have led to recent increase in numbers of river otters and their geographical distribution [1, 2]. In regions where otters have been present for longer periods of time [3–5], the species’ diet varies considerably with geographic region. However, little is known about how this diet varies in regions where otters have been recently reintroduced. Furthermore, information about diets have generally been obtained by examining gut or fecal contents, which relies primarily on the identification of hard or bony tissues. These methods underestimate the contribution of dietary items without such hard tissues, such as molluscs and insect larvae [6, 7]. In regions where prey species may be endangered, wildlife management agencies need to better understand the potential impact of otter predation on these species to better protect them.

To compensate for the limitations in earlier methods, diet can also be inferred from fatty acid analysis. For example, quantitative fatty acid signature analysis (QFASA [8]) is a technique capable of providing high resolution in differentiating diet species. Unlike fecal and gut-content analyses, tissue fatty acids (FA) reflect diet over a longer time span, and a predator’s FA profile is a proportionate representation of its prey. QFASA has been used to determine the diet of marine mammals, including cetacean [9–11] and pinniped species [8, 12, 13], and is based on: (1) variable ability to metabolize and store FAs depending on chain length and saturation, and (2) concentration of unusual FA up the food chain. Experiments with harbor seals in Prince William Sound, Alaska demonstrated that QFASA was able to determine the specific fish species in the diet of seals, and reflect differences in diet with age and fine-scale habitat [13]. Because different FAs may be metabolized differently, the FA profile of the predator’s diet may not match the FA profile of the predator itself. Nevertheless, experimental studies indicate that QFASA has a high rate (88%) of correctly inferring diets when diets were known [14].

Our primary objective in this paper was to determine how well differences in fatty acid signatures could be used to identify different taxonomic groups among prey species that river otters are known or suspected to consume. We hypothesized that differences in fatty acid profiles would allow for the identification of large taxonomic groups (fishes, crayfish, amphibians, and molluscs), and possibly lower-level taxonomic distinctions of animals being consumed by otters. We demonstrate that the different taxa considered have significantly different FA profiles, and provide the otter diet as inferred by QFASA.

## Methods

### Species and tissues analyzed

All animals included in this analysis were from Illinois, USA. The Illinois Department of Natural Resources provided us with river otters obtained from incidental deaths including road kills and unintended trapping. Otter carcasses were stored frozen until transport to our laboratory. All otter carcasses were gathered between 21 Oct and 15 Mar, with 12 (26%) obtained in the fall (Oct, Nov), 26 (57%) in the winter (Dec-Feb), and 8 (17%) in the spring (Mar). Further details on the otters sampled are available in Carpenter et al. [15]. Crayfish samples were collected Feb-Mar, and fish, amphibian, and mollusc samples between Feb-May.

River otter adipose tissue was dissected from two deposits: dorsal subcutaneous adipose from the ventral side of the base of the tail, approximately 5 cm posterior of the anus (*n* = 46), and from the footpads of the forefeet (*n* = 19). Lipid was extracted separately from each the two adipose deposits. A smaller number of samples were analyzed from the footpads, as these were intended only to verify the assumption that this deposit would be cold-adapted as suggested in Käkelä and Hyvärinen [16].

Species that were potential prey for otters, including 15 fish species, 5 mollusc species, 3 amphibian species, and 2 species of crayfish (Table 1) were obtained opportunistically from colleagues at the Illinois Natural History Survey, in conjunction with their individual research agendas. For all prey species, whole-body homogenate of the entire specimen, excluding shells for molluscs, was used for lipid extraction.

**Table 1:** **River otter and its candidate prey species evaluated in the study, Illinois, USA**

Taxonomic division	Scientific name
Carnivores (predator species)	Nearctic River Otter *Lontra canadensis* (*n* = 46; 46 tail, and 19 footpad samples)
Fishes (prey)	
Clupeiformes	Gizzard Shad *Dorosoma cepedianum* (*n* = 6)
Cypriniformes	Asian Carp^a^ *Hypophthalmichthys nobilis* (*n =* 5)
	Hornyhead Chub *Nocomis biguttatus* (*n* = 3)
	Creek Chub *Semotilus atromaculatus* (*n* = 7)
Perciformes	Greenside Darter *Etheostoma blennioides* (*n =* 6)
	Bluegill *Lepomis macrochirus* (*n* = 10)
	Redear Sunfish *Lepomis microlophus* (*n* = 9)
	Smallmouth Bass *Micropterus dolomieu* (*n* = 7)
	Largemouth Bass *Micropterus salmoides* (*n* = 6)
	White Crappie *Pomoxis annularis* (*n* = 1)
	Black Crappie *Pomoxis nigromaculatus* (*n* = 4)
Siluriformes	Black Bullhead *Ameiurus melas* (*n* = 8)
	Blue Catfish *Ictalurus furcatus* (*n =* 8)
	Channel Catfish *Ictalurus punctatus* (*n =* 8)
	Brindled Madtom *Noturus miurus* (*n* = 2)
Molluscs (prey)	Threeridge *Amblema plicata* (*n =* 2)
	Asian Clam *Corbicula fluminea* ^a^ (*n* = 10)
	Wabash Pigtoe *Fusconaia flava* (*n =* 10)
	Fat Mucket *Lampsilis siliquoidea* (*n* = 10)
	Round Pigtoie *Pleurobema sintoxia* (*n =* 5)
Amphibians (prey)	Cricket Frog *Acris crepitans* (*n* = 10)
	Western Chorus Frog *Pseudacris triseriata* (*n* = 1)
	American Bullfrog *Rana catesbeiana* [*Lithobates catesbeianus*] (*n* = 1)
Crayfish (prey)	Northern Clearwater Crayfish *Orconectes propinquus* (*n =* 10)
	Virile Crayfish *Orconectes virilis* (*n =* 4)

^a^Invasive species.

### Gas–liquid chromatography

Lipids were extracted from tissue or homogenates with choloroform:methanol (2:1 by volume) by mixing in a Polytron for 30 seconds [17]. The chloroform phase containing lipid was removed, the extraction repeated twice, and chloroform evaporated under a stream of nitrogen at room temperature. FA methyl esters were prepared as described by AOCS official method Ce 2–66 [18]. Subsequently, FA methyl esters were analyzed using a gas chromatograph (Hewlett Packard 5890 series II) with a DB-wax capillary column (30 m × 0.25 mm × 0. 25 μm film coating, Agilent Technologies, Santa Clara, CA). The column was under a constant pressure at 1.30 kg/cm^2^ using helium as the carrier gas. Temperature of the injector and of the flame-ionization detector was held constant at 250°C and 260°C, respectively. The oven was operated at 170°C for 2 min (programmed temperature to increase 2°C /min up to 240°C and then held constant for 10 min). Chromatographs from FA methyl esters were integrated using Agilent Chemstation software for gas chromatographs systems (Version B.01.02, Agilent Technologies, Inc.®). We identified FA methyl esters by comparing retention times of known standards (GLC 461A, Nu-check-prep, Elysian, MN). Some FA peaks did not correspond to the 30 fatty acids present in this standard. We estimated the total number of fatty acids (60) based on the number previously reported for several species [16], and assigned identity of unknown peaks by running the k-means clustering method in R (version 2.15.0) [19, 20] to account for variation in retention times between runs. Centroids for unidentified peaks were randomly initialized, with a uniform distribution between the minimum and maximum observed retention-time values. We repeated the process of initializing centroids and grouping peaks by the k-means algorithm 1000 times. For each run, variance explained by the clustering (SS_between clusters_/SS_total_) was recorded and the run that explained the greatest amount of the variance was used to determine the best-fit assignment of unknown peaks.

### Principal components analysis

Principal components analysis (PCA) is an analytical method that compresses multiple variables into a more tractable number of linearly uncorrelated variables, while simultaneously maximizing the amount of variance explained by the new, compressed and lower-dimensional set of variables.

The FA profile of each individual animal was comprised of 60 different FA. The proportion of each individual FA was treated as a variable and PCA was conducted on otters and prey. An analysis was performed on otter adipose deposits to determine if fatty acid profiles were different for subcutaneous and footpad adipose. Individual PCA were also performed on all samples within each taxonomic group (crayfish, frogs, fishes, and molluscs) and on species within a given taxonomic group to determine if PCA could be used to identify individual species on the basis of their fatty acid profiles. All analyses were conducted using the FactoMineR library for R (described in [21]).

For each PCA, we performed an analysis of variance (ANOVA) on the first several principal components to determine significant differences of mean contributions to each component. For the PCA including all samples, the factors were the large taxonomic divisions (otter, mollusc, fish, crayfish, frog). For PCA within each subdivision, the factors were individual species, and for the PCA on otter adipose only, the factors were the two deposits—tail and footpad. The *p* values for all ANOVA were corrected using Tukey’s Honest Significant Differences.

### Diet inference—quantitiative fatty acid signature analysis

We estimated the contribution of each taxonomic group to the otter diet using the quantitative fatty acid signature analysis method described in Iverson et al. [8]. Means and standard errors for each proportion are based on 500 bootstrapped samples.

## Results and discussion

**Figure 1:**
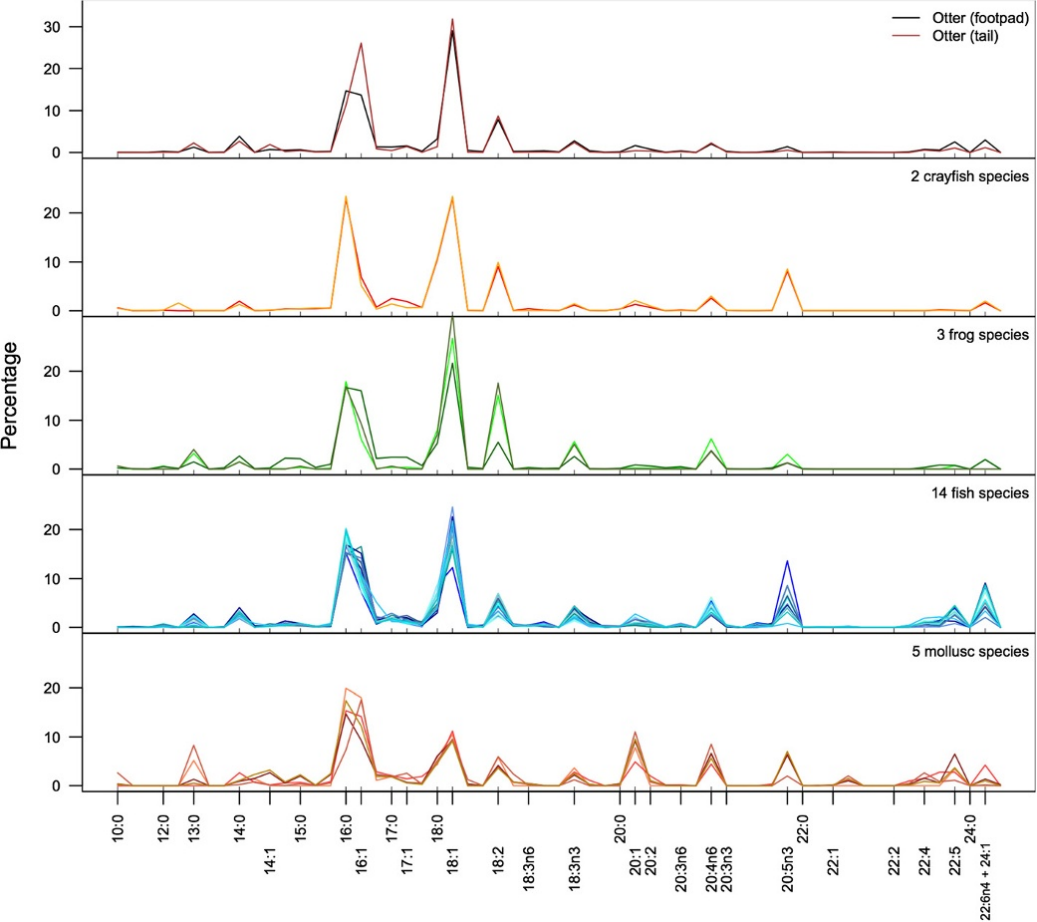
**Fatty acid distributions in five taxonomic groups in Illinois, USA.** Lines represent fatty acid profiles, and are not chromatograms. The distributions of fatty acids in Illinois river otters (tail: *n* = 46; footpad: *n* = 19), and 2 crayfish species (*n* = 14), 3 frog species (*n* = 12), 14 fish species (*n* = 56), and 5 mollusc species (*n* = 37). Each line indicates the mean value for all individuals of a given species. The y-axis is the percentage makeup by mass. All fatty acids were assigned to one of 60 possible groups. Those which we were able to identify from our standard are labeled as tics in each graph, and with their identity presented along the x-axis.

In both otters and their potential prey species, including, fishes, frogs, crayfish, and molluscs, 16:0, 16:1, 18:0, and 18:1 were the predominate FA (Figure 1). This is not surprising, as these four fatty acids predominate in most animals [22]. Across all samples, these four FA comprised a mean of 55.9% (±9.2% sd) of the total FA. Within these four FA, however, molluscs and crayfish differed from the vertebrate animals. Molluscs had higher proportions of 16:0 and 16:1, whereas vertebrate taxa had higher proportions of 18:0 and 18:1. Crayfish have approximately equal proportions of 16 and 18-carbon FA. These four FA were important in PCA because of their predominance in most species. However, the remaining FA were particularly important for identifying taxon-specific variation. For example, considerable variation is evident in the long-chain FA, particularly the 20-carbon FA (20:0 – 20:5n3). Fish, mollusc, and crayfish species all showed higher proportions of omega-3 FA (20:5n3) than frogs or otters; molluscs, fish, and frog species had higher proportions of omega-6 (20:4n6) than otters or crayfish. Longer chain FA (22-C and above) were in low proportions in all taxa other than fishes and molluscs. A summary of the fatty acid signatures for all species is provided in Table 2.

**Table 2:** **Fatty acids of the river otter (** ***Lontra canadensis*** **) and several candidate prey species; Illinois, USA**

FA	Otter		Crayfish	Frog	Mollusc	Fish^a^			
	Tail	Footpad				Clup.	Cypr.	Perc.	Silur.
10:0	0.0 ± 0.03^b^	0.0 ± 0.02	0.5 ± 0.42	0.2 ± 0.19	0.4 ± 1.47	0.1 ± 0.04	0.1 ± 0.08	0.1 ± 0.06	0.0 ± 0.05
12:0	0.2 ± 0.10	0.1 ± 0.07	0.1 ± 0.10	0.6 ± 0.51	0.0 ± 0.01	0.1 ± 0.02	0.2 ± 0.21	0.3 ± 0.26	0.5 ± 0.57
13:0	1.3 ± 2.09	2.3 ± 3.41	0.0 ± 0.00	3.1 ± 3.51	1.7 ± 3.61	0.3 ± 0.30	1.3 ± 1.73	1.0 ± 1.78	1.1 ± 2.25
14:0	3.8 ± 1.05	2.7 ± 0.99	1.5 ± 0.64	1.6 ± 0.47	1.3 ± 0.85	4.1 ± 0.81	2.5 ± 0.96	2.7 ± 0.64	2.5 ± 1.06
14:1	0.7 ± 0.45	1.9 ± 1.04	0.1 ± 0.14	0.2 ± 0.15	1.6 ± 1.95	0.2 ± 0.03	0.4 ± 0.25	0.4 ± 0.25	0.3 ± 0.18
15:0	0.6 ± 0.25	0.4 ± 0.24	0.4 ± 0.19	0.5 ± 0.55	1.3 ± 1.13	0.8 ± 0.24	0.6 ± 0.15	0.6 ± 0.23	0.6 ± 0.11
16:0	14.7 ± 3.33	11.3 ± 5.91	23.3 ± 8.09	17.7 ± 1.6	15.6 ± 4.08	17.0 ± 3.33	15.9 ± 1.98	17.7 ± 2.57	18.9 ± 1.73
16:1	13.7 ± 4.65	26.1 ± 9.01	5.7 ± 2.94	7.1 ± 3.38	13 ± 4.05	15.2 ± 7.16	10.2 ± 3.29	12.4 ± 3.23	8.9 ± 2.77
17:0	1.3 ± 0.47	0.4 ± 0.35	1.7 ± 0.89	0.5 ± 0.63	1.9 ± 0.44	1.9 ± 0.39	1.7 ± 0.46	1.9 ± 0.62	1.8 ± 0.51
17:1	1.6 ± 0.40	1.4 ± 0.51	1.0 ± 0.92	0.5 ± 0.65	1.0 ± 0.83	2.0 ± 0.47	1.2 ± 0.87	1.5 ± 0.73	0.9 ± 0.29
18:0	3.2 ± 1.60	1.4 ± 0.69	10.7 ± 5.57	7.7 ± 2.24	5.2 ± 1.20	3.0 ± 1.32	6.9 ± 2.59	4.8 ± 1.35	7.7 ± 1.76
18:1	29 ± 4.80	31.9 ± 8.71	23.3 ± 5.33	26.7 ± 3.38	9.8 ± 1.37	22.6 ± 5.28	19.8 ± 5.93	18.2 ± 2.96	20.1 ± 2.64
18:2	7.9 ± 3.30	8.7 ± 3.70	9.7 ± 4.12	14.6 ± 3.25	4.3 ± 0.99	3.3 ± 0.33	5.2 ± 2.63	5.4 ± 1.79	3.2 ± 1.74
18:3n6	0.3 ± 0.10	0.1 ± 0.12	0.2 ± 0.26	0.1 ± 0.17	0.3 ± 0.26	0.4 ± 0.06	0.4 ± 0.13	0.4 ± 0.11	0.2 ± 0.07
18:3n3	2.8 ± 0.89	2.4 ± 1.25	1.4 ± 1.37	5.4 ± 4.83	2.5 ± 0.72	3.9 ± 0.62	2.4 ± 1.29	3.4 ± 1.47	1.8 ± 0.76
20:0	0.1 ± 0.07	0.0 ± 0.03	0.3 ± 0.10	0.1 ± 0.13	0.3 ± 0.20	0.2 ± 0.06	0.2 ± 0.11	0.2 ± 0.09	0.3 ± 0.04
20:1	1.6 ± 0.73	0.4 ± 0.37	1.9 ± 1.54	0.2 ± 0.26	8.8 ± 2.98	0.8 ± 0.20	1.5 ± 0.70	1.0 ± 0.91	1.3 ± 0.77
20:2	0.8 ± 0.24	0.3 ± 0.27	0.9 ± 0.42	0.2 ± 0.19	1.2 ± 1.27	0.7 ± 0.21	0.7 ± 0.41	0.5 ± 0.44	0.5 ± 0.28
20:3n6	0.4 ± 0.10	0.2 ± 0.20	0.1 ± 0.12	0.3 ± 0.26	0.1 ± 0.10	0.3 ± 0.07	0.6 ± 0.42	0.3 ± 0.17	0.4 ± 0.11
20:4n6	2.0 ± 0.8	2.3 ± 1.22	2.9 ± 1.31	5.8 ± 2.33	5.8 ± 1.66	2.5 ± 0.92	4.7 ± 2.10	3.6 ± 1.22	5.0 ± 1.90
20:3n3	0.3 ± 0.09	0.1 ± 0.13	0.1 ± 0.16	0.1 ± 0.14	0.0 ± 0.05	0.1 ± 0.03	0.2 ± 0.11	0.3 ± 0.19	0.2 ± 0.07
20:5n3	1.4 ± 0.63	0.5 ± 0.37	8.4 ± 3.83	2.8 ± 0.83	5.8 ± 2.00	4.6 ± 0.86	7.8 ± 4.49	4.3 ± 2.72	6.1 ± 1.36
22:0	0.0 ± 0.02	0.0 ± 0.02	0.0 ± 0.03	0.0 ± 0.04	0.1 ± 0.54	0.0 ± 0.04	0.0 ± 0.00	0.1 ± 0.06	0.1 ± 0.06
22:1	0.1 ± 0.06	0.0 ± 0.01	0.0 ± 0.02	0.0 ± 0.01	0.2 ± 0.45	0.1 ± 0.05	0.0 ± 0.02	0.1 ± 0.06	0.0 ± 0.05
22:2	0.0 ± 0.01	0.0 ± 0.00	0.0 ± 0.00	0.0 ± 0.00	0.0 ± 0.05	0.0 ± 0.02	0.0 ± 0.00	0.0 ± 0.02	0.0 ± 0.02
22:4	0.7 ± 0.25	0.6 ± 0.51	0.0 ± 0.00	0.1 ± 0.15	1.3 ± 0.69	0.4 ± 0.23	0.4 ± 0.38	0.9 ± 0.65	1.0 ± 0.57
22:5	2.5 ± 0.88	1.1 ± 0.98	0.0 ± 0.13	0.6 ± 0.54	3.7 ± 2.09	1.3 ± 0.25	2.2 ± 1.59	3.3 ± 1.32	3.0 ± 0.57
24:0	0.0 ± 0.01	0.0 ± 0.00	0.0 ± 0.00	0.0 ± 0.00	0.0 ± 0.04	0.0 ± 0.02	0.0 ± 0.00	0.1 ± 0.09	0.1 ± 0.08
22:6n4 + 24:1^c^	3.0 ± 1.11	1.2 ± 1.06	1.9 ± 0.92	1.8 ± 1.27	1.8 ± 1.56	4.3 ± 0.77	6.2 ± 2.59	6.8 ± 2.72	6.4 ± 2.41

^a^Because a relatively large number of fish species were included in this study, they are subdivided here by order: Clupeiformes, Cypriniformes, Perciformes and Siluriformes (see Table 1).

^b^All values given as percentage of total fatty acids by mass ± 1sd.

^c^The column used in gas chromatography did not separate 22:6n4 and 24:1 and therefore they were grouped together.

### Principal components analysis

#### Prey species by taxonomic group

**Figure 2:**
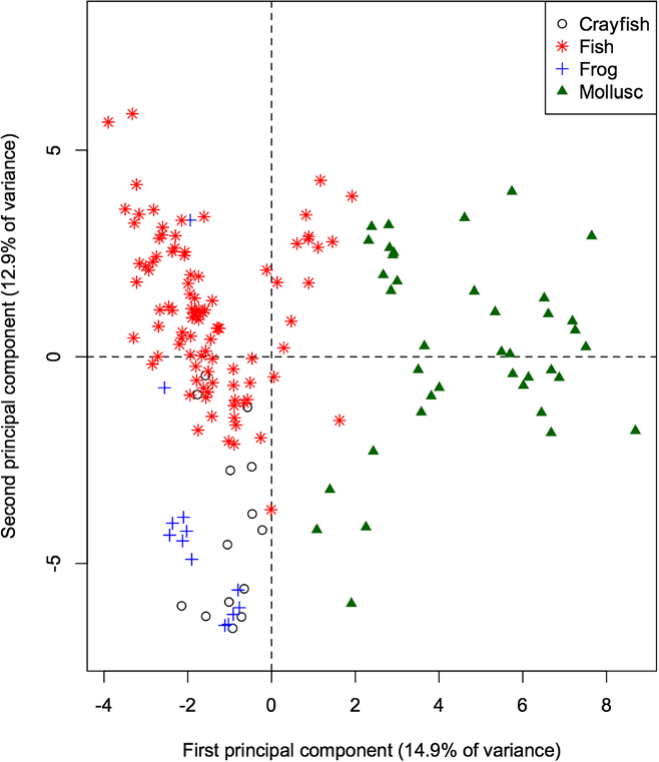
**Principal components analysis of fatty acid in four major taxonomic groups that are potential prey species for Illinois river otters.** Each data point represents the principal components of 60 FA from an individual animal, jointly accounting for 27.8% of the variance in FA. There is considerable variation between individuals but taxonomic groups (identified by color) form identifiable clusters on the plot so that mollusc, fish, and frog species can be distinguished from each other. Crayfish occupy the space between frogs and fishes.

The 60-dimensional FA signature of all prey species projected onto the first 2 principal components (Figure 2) provides a quick visual interpretation of overall similarity between fatty acid signatures, as more similar profiles will be projected more closely together. The first PC accounts for 14.9% of the total variation in FA in all potential prey species, and separates the molluscs from the remaining taxonomic groups (ANOVA: *p* < 0.001 for all pairwise differences with molluscs). Mean contributions to the component also separated frogs and crayfish (*p* = 0.011). This component is dominated by relatively high proportions of 20:1, and relatively low proportions of 18:1, 14:0, and 20:3n3 in molluscs. These values can be quantified by how strongly the proportion of each FA correlates with the component. Specifically, 20:1 shows a strong positive correlation (0.89) with the first PC, whereas 18:1, 14:0, and 20:3n3 have strong negative correlations (−0.66, −0.62, and −0.53 respectively).

The second PC accounts for an additional 12.9% of the total variation, and separates fish species from frogs and crayfish (both *p* < 0.001), contrasting relatively higher proportions of 17:1 (correlation = 0.60) and 16:1 (0.60) in fishes, and relatively low proportions of 18:0, 18:2, and 10:0 (correlations = −0.64, −0.52, and −0.44 respectively).

#### Comparing species within a single taxonomic group

**Figure 3:**
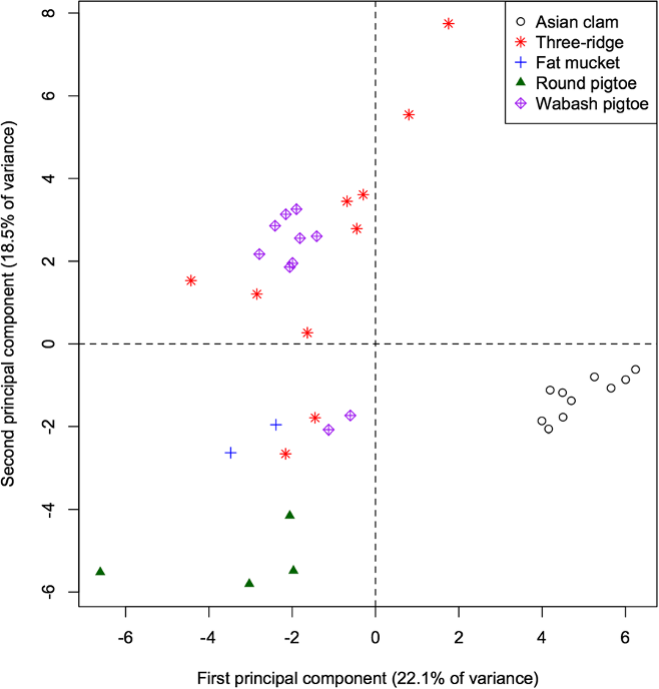
**Principal components analysis of fatty acids in five mollusc species in Illinois.** The data from 60 unique fatty acids projected onto the first two principal components (jointly accounting for 40.6% of the total variance in their fatty acid profiles). Asian clams (*Corbicula fluminea*, an invasive species) and the round pigtoe (*Pleurobema sintoxia*) form clusters, whereas the three-ridge (*Amblema plicata*), Wabash pigtoe (*Fusconaia flava*), and fat mucket (*Lampsilis siliquoidea*), have fatty acid profiles that form less distinct clusters with some overlap between individuals.

Analysis of fatty acid signatures within the 5 mollusc species again yielded apparent clusters (Figure 3). The first PC accounts for 22.1% of the total variation among mollusc FA signatures and separates the invasive Asian clam (*Corbicula fluminea*) from the native species (*p* < 0.001 for all pairwise differences), as well as separating the round pigtoe (*Pleurobema sintoxia*) from the fat mucket (*Lampsilis siliquoidea*; *p* = 0.045). This component is characterized by relatively high proportions of 10:0, 20:1, and 13:0 in native species (correlations = −0.57, −0.55, −0.46).

The second PC accounts for an additional 18.5% of the total variation in molluscs, and separates the round pigtoe (*Pleurobema sintoxia*) from both the fat mucket and the Wabash pigtoe (*Fusconaia flava*; both *p* < 0.001), as well as further separating the round pigtoe from the Asian clam (*p* = 0.019). This component is characterized by relatively high proportions of 17:1 and 16:1 (correlations = 0.63, 0.60) in *Pleurobema*, and low proportions of 14:1 and 22:1 (correlation = 0.70 for both). The 4^th^ PC (not shown) separates the Wabash pigtoe from the fat mucket (*p* = 0.004). The first 5 PC all failed to separate the three ridge (*Amblema plicata*) from either the round pigtoe or the Wabash pigtoe (all *p* > 0.05).

Other taxonomic divisions had similar results. The two crayfish species differed in their mean contributions to the 3^rd^ PC (*p* = 0.011). Among frogs, both *Acris* and *Pseudacris* differed from *Lithobates* along the 1^st^ PC (*p* = 0.003 and 0.010 respectively), and from one another along the 5^th^ PC (*p* < 0.001). Among the fish species, 87% of the pairwise differences were significant (*p* < 0.05) on at least one of the first 5 PC—higher PC were not analyzed.

#### River otter fatty acids by fat deposit

**Figure 4:**
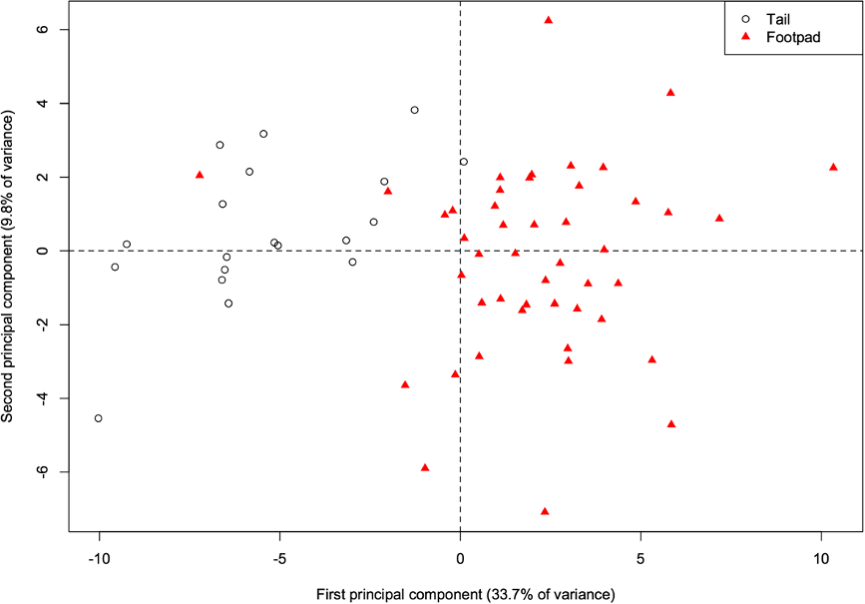
**Principal components analysis of fatty acids in Illinois river otters.** The data from 60 unique fatty acids projected onto the first two principal components (jointly accounting for 27.8% of the total variance in otter fatty acid profiles). Each data point represents a tissue sample from a single animal. Footpad and tail deposits have largely distinct fatty acid profiles, as there is very little overlap between the two clusters.

Within our river otter samples, fatty acid profiles differed according to the deposit from which the adipose sample was dissected (either from the base of the tail or the footpad of the forepaw). The 1^st^ PC alone accounts for 33.7% of the total variance, and there is already considerable separation (*p* < 0.001) of the two deposits along this component (Figure 4). This component is primarily characterized by higher proportions of 20:1, 20:0, and 17:0 in the tail (correlations = 0.87, 0.86, 0.79), and higher proportions of 16:1 and 14:1 (−0.84, −0.79) in the footpad. More generally, the footpad deposit is characterized by lower concentrations of saturated fats. With the exception of 13:0, all other saturated fats are positively correlated with this component, indicating higher proportions in the tail (correlations for 10:0, 12:0, 14:0, 15:0, 16:0, 17:0, 18:0, 20:0, 22:0, and 24:0 are 0.34, 0.75, 0.39, 0.62, 0.26, 0.79, 0.57, 0.86, 0.25, and 0.29 respectively). In a study of several species, Käkelä and Hyvärinen [16] demonstrate similarly high concentrations of unsaturated FA in the extremities of cold-adapted species. The FA signature of the footpads is therefore likely to be cold-adapted as well, and thus a biased indicator for inferring diet. Thus, when analyzing the otter FA signatures relative to the other species, we included only the adipose tissue from the base of the tail, with the assumption that this depot did not suffer the same bias.

#### Otter and potential prey combined

**Figure 5:**
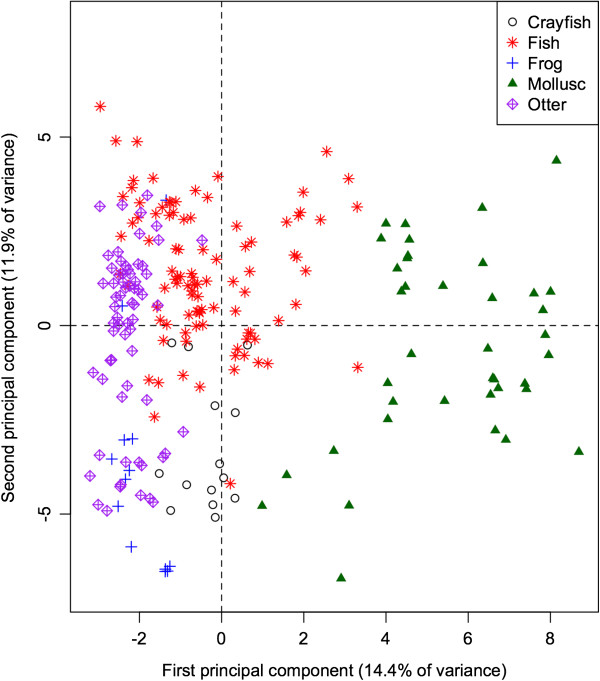
**Principal components analysis of 60 fatty acids in Illinois river otters (** ***Lontra canadensis*** **) and several of its candidate prey species.** Of the candidate prey species considered here, the river otter fatty acid signatures are most similar to those of fish species as indicated by their proximity in the graph. This suggests that fish are the predominant component of the otter diet, though a better understanding of how river otters metabolize different fatty acids is necessary for a fuller inference of the PCA results for diet.

Adding the otter data to the PCA of its potential prey species (Figure 5) indicates that the otters have fatty acid signatures that are most similar to fish species. Like fish and frogs, the proportions of the 18-carbon chains are higher than those of 16-carbon chains. Similarly, like fish, and unlike frogs, otters show higher proportions of the long-chain FA (particularly 24:0) than the other taxa.

#### Estimation of the otter diet

The proportions of each taxon were estimated at 37.7 ± 1.0% (SE) fishes, 32.0 ± 0.8% mollusc, 27.3 ± 4.3% amphibian, and 3.0 ± 0.6% crayfish by mass.

#### Discussion

Large-scale taxonomic differences were discernible from FA signatures. Species-level differences were largely significant as well, though not universally. Within the mollusc and fish divisions, some species did not differ significantly on any of the first 5 principle components. Where significance was not demonstrable, it may have been due to small sample sizes, but even with such small sample sizes, the majority of pairwise differences were significant. These results provide further evidence that FA signatures are capable of demonstrating fine-grain differences between species, and potentially diet. Nevertheless, we recognize a number of limitations in interpreting these results. In particular, it is possible that FA signatures for a given species may show significant variation both seasonally and geographically, as both may lead to differences in availability of prey that are sufficient to change the species’ FA makeup. In using FA signatures to infer diet, it is necessary to determine the degree to which individual species or taxonomic groups may be discerned from each other on the basis of their FA signatures as we have done here. However, this information alone is insufficient to make more than general inferences about the diet. In order to make more accurate inferences, further information is needed regarding how the predator species metabolizes different FA. The QFASA adjusts for this shortcoming by measuring the distance between the predator (otter) FA profile and that of the diet by using the Kulback-Liebler (KL) distance (in [8]), which gives greater weight to rare FA. When comparing results among different distance metrics, including the usual squared error, squared relative error, the squared error distance of logs, and the KL on a controlled diet, KL was shown to perform well and is a natural distance metric for comparing distributions [8].

Throughout the results above, we reported differences only for those FA that we were able to identify from the standard. However, variations also existed in the unidentified FA but since the FA were unidentified, we omitted this information.

## Conclusion

We demonstrate that there are taxon-specific differences in fatty acid signatures that allow for the unique identification of taxonomic groups (Figure 2). In previous studies, QFASA has been largely successful at inferring diets in marine mammals. One study on harbor seals even inferred the geographic location where predation had occurred, and the average size of the fish being preyed upon on the basis of unique FA signatures [13]. However, river otters have been reported to prey on a greater variety of species, including, most commonly, a wide variety of fish species, crayfish, and frogs, but have also been reported to eat other reptiles and amphibians—including snakes, salamanders, and turtles—molluscs, insects and insect larvae, and occasionally birds and other mammals. The greater diversity of prey species results in a greater likelihood that prey species will have overlapping (not uniquely identifiable) FA signatures, making it more difficult to accurately infer the diet.

The findings in this paper suggest that large-scale taxonomic differences (i.e., the relative contributions of fish, crayfish, frog, or mollusc) in the river otter diet have significantly different FA profiles, and the QFASA estimates similarly have small standard errors. Many finer-scale differences such as individual species may also be discernible with sufficiently large samples, though the PCA analyses indicate that not all species will be discernible from FA signatures alone. The analyses indicated which species are likely to have the most similar signatures, such as the mollusc species, the three ridge, the round pigtoe, and the Wabash pigtoe, none of which had significantly different signatures along any of the first five PC.

The results of this study provide an initial inference of the otter diet. This inference is limited, and a more accurate inference of the diet requires knowledge of how otters metabolize different FA. However, experimental studies with controlled diet have demonstrated that QFASA has a high rate of accurately inferring diet [14], though this accuracy may diminish with the complexity of the diet. Although a more accurate inference may be gained by studying fat metabolism, such studies are costly, and for many species such as otters and many marine species, capturing and restraining the animals in order to perform the studies is often difficult or impossible. Thus, although the inference is limited, these limitations are counterbalanced by the relative ease and lower cost of the methods described herein. Furthermore, it is important to recognize that, molluscs, which have generally been absent from previous otter diet studies, presumably because remains do not show up in fecal samples, were estimated here to constitute as much as 32% of the otter diet in Illinois. In regions where mollusc species are endangered, these findings have important implications for wildlife management.

This study did not analyze all candidate prey taxa. In particular we did not analyze insect, mammal, or avian fatty acid profiles, but instead focused on those species most commonly reported in the river otter diet. The results here must therefore be considered the upper limits of the proportion of each taxon in the Illinois river otter diet. Furthermore, there is likely to be some seasonal variation in the otter diet, and none of our otter specimens were obtained during the summer months (Jun-Aug).

## Abbreviations

ANOVA: Analysis of variance
AOCS: American Oil Chemists’ Society
FA: Fatty Acid(s)
GLC: Gas–liquid Chromatography
PC: Principle Component(s)
PCA: Principal Components Analysis
QFASA: Quantitative Fatty Acid Signature Analysis
SS: Sum of Squares
USA: United States of America.
